# A novel method to make viscoelastic polyacrylamide gels for cell culture and traction force microscopy

**DOI:** 10.1063/5.0002750

**Published:** 2020-07-02

**Authors:** Elisabeth E. Charrier, Katarzyna Pogoda, Robin Li, Chan Young Park, Jeffrey J. Fredberg, Paul A. Janmey

**Affiliations:** 1 Institute for Medicine and Engineering, University of Pennsylvania, Philadelphia, Pennsylvania 19104, USA; 2 Integral Molecular, Philadelphia, Pennsylvania 19104, USA; 3 Institute of Nuclear Physics Polish Academy of Sciences, PL-31342 Krakow, Poland; 4 Department of Environmental Health, Harvard T.H. Chan School of Public Health, Boston, Massachusetts 02115, USA; 5 Department of Physiology, University of Pennsylvania, Philadelphia, Pennsylvania 19104, USA

## Abstract

Polyacrylamide hydrogels are commonly used in cell biology, notably to cultivate cells on soft surfaces. Polyacrylamide gels are purely elastic and well adapted to cell culture as they are inert and can be conjugated with adhesion proteins. Here, we report a method to make viscoelastic polyacrylamide gels with mechanical properties more closely resembling biological tissues and suitable for cell culture *in vitro*. We demonstrate that these gels can be used for traction force microscopy experiments. We also show that multiple cell types respond to the viscoelasticity of their substrate and that viscous dissipation has an influence on cell spreading, contractility, and motility. This new material provides new opportunities for investigating how normal or malignant cells sense and respond to viscous dissipation within the extra-cellular matrix.

## INTRODUCTION

Polyacrylamide (PAA) hydrogels are widely used to study *in vitro* the effect of the mechanical environment on cell behavior.^1–4^ PAA gels are nearly purely elastic, with the shear storage modulus several orders of magnitude larger than the loss modulus. The storage modulus of the gel is determined by the concentration of polymerized acrylamide and the degree of cross-linking. Over the last two decades, protocols have been optimized to tune PAA gels for a range of different stiffnesses suitable for cell culture.^5^ The use of these materials has shed light on the involvement of the cellular microenvironment in controlling cell motility, cell cycle, spreading, and differentiation.^3,4,6–8^ In contrast to PAA gels, which are elastic, soft biological tissues are viscoelastic^9–15^ and, thus, characterized by both a storage and an appreciable loss modulus. Until recently, only the storage modulus of tissues could be reproduced by classical PAA gels *in vitro*. In order to reproduce more precisely the mechanical properties of biological tissues, we developed a method to make viscoelastic PAA gels that reproduce both the storage and loss moduli of biological tissues.^16^

As previously described, viscoelastic PAA gels are composed of two types of polyacrylamide: a covalently crosslinked PAA network and long, entangled polyacrylamide molecules, which are trapped within the PAA network.^16^ This unique formulation leads to the formation of a hydrogel that is mechanically described as a viscoelastic solid. We have previously reported the measurement of viscoelasticity of crosslinked polyacrylamide, and our reports are consistent with the large body of literature reporting that crosslinked polyacrylamide gels are nearly ideal linear elastic materials.^16–19^ Linear elasticity implies that the shear modulus is independent of strain, and this feature facilitates the ability to calculate traction stresses from the measured strain field. This manuscript describes the step-by-step protocol to make these viscoelastic PAA gels and functionalize their surface with adhesion proteins. We then demonstrate that these hydrogels are suitable for traction measurements and that the average contractile force of NIH 3T3 fibroblasts is reduced on viscoelastic gels, while their motility is increased. We also show that multiple cell types respond to viscous dissipation. Altogether, our observations suggest that viscoelastic polyacrylamide gels are a promising system to maintain primary or stem cells in culture under approximately physiological mechanical conditions as well as to study the role of viscous dissipation in the cell phenotype. Additionally, these matrices are novel tools to study the consequences of changes in viscous dissipation, which occur during disease progression on cell and tissue function.

## RESULTS AND DISCUSSION

To define the effect of viscous dissipation on cells from different histological origins, multiple cell types were plated on viscoelastic gels. We examined cell spreading, motility, and contractility.

### Viscous dissipation affects fibroblast motility and contractility

We have previously reported that when collagen I is presented on the PAA network, 3T3 fibroblasts spread less on viscoelastic gels than on elastic gels.^16^ However, when collagen I is crosslinked to both types of PAA, fibroblasts spread similarly on elastic and viscoelastic gels.^16^ In order to have a better understanding of the role of viscous dissipation in the regulation of cell contractility, we quantified the contractile moment of 3T3 fibroblasts by traction force microscopy.^22^

**FIG. 1.**
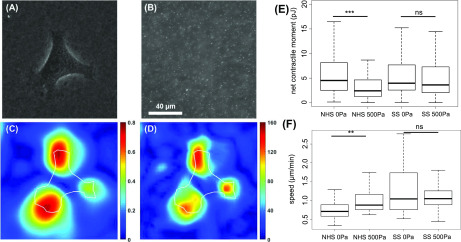
Traction measurement of 3T3 fibroblast on viscoelastic PAA gel. Dispersion of fluorescent beads in the gel, motility, and contractile energy of 3T3 fibroblasts plated for 24 h on elastic and viscoelastic PAA gels.

0.2 *μ*m diameter fluorescent beads were incorporated into the gel in order to analyze its deformation due to the presence of cells on the gel. The fluorescent beads are well dispersed in both the elastic and the viscoelastic gels, allowing us to calculate the strain fields within the hydrogel surface (Fig. 1). The contractility of 3T3 cells has been characterized by the traction force microscopy method previously described.^24^ On gels presenting collagen I on the network of PAA, fibroblasts are less contractile on viscoelastic gels than on elastic gels (Fig. 1). We point out that 3T3 cells do not form stress fibers on viscoelastic gels presenting collagen I only on the network, which might hinder cell force generation. When collagen I is presented on both forms of PAA, the contractile moment of 3T3 cells is similar on elastic and viscoelastic gels. This observation demonstrates that dissipating part of the cellular energy through the substrate is not sufficient to reduce cell contractility, as when collagen I is presented through both types of PAA, the contractile moment of 3T3 fibroblasts is similar on elastic and viscoelastic gels of similar storage modulus. These results suggest that the average contractile moment of 3T3 cells is related to the average cell area reported in the study by Charrier *et al.*,^16^ which is not surprising as widely spread cells form focal adhesions and stress fibers^16,25^ that produce and transmit the cellular forces to the extracellular matrix.^26,27^ It is worth noting that the cellular response to viscous dissipation can be different for different cell types, and specifically, it can be altered in cancerous and highly malignant cells. Recent studies of Huh7 hepatocellular carcinoma cells showed that despite having a larger cellular area on viscoelastic gels compared to elastic ones, the cell traction stresses generated by these cells are comparable on elastic and viscoelastic substrates.^19^ Inhibition studies by Mandal *et al.*^19^ with latrunculin A and withaferin A have shown that actin and vimentin inhibition restricted cell spreading more on viscoelastic than elastic substrates, but further studies are needed to reveal underlying mechanisms. This result suggests that dissipative systems need to be tested with many other normal and pathologically altered cell lines to fully understand the cell mechanoresponse in normal and diseased tissues.

(A) Phase image of the 3T3 fibroblast cell on viscoelastic (500 Pa) gel.
(B) Fluorescence images of 0.2 *μ*m beads inside the viscoelastic (500 Pa) gel.
(C) Deformation image of the viscoelastic (500 Pa) gel in *μ*m.
(D) Calculated traction stress in Pa.
(E) Average net contractile moment of 3T3 fibroblasts on elastic (G″ ∼ 0 Pa) and viscoelastic (G″ = 500 Pa) gels presenting collagen I on the network of PAA (NHS) or on both types of PAA (SS).
(F) Average cell speed of 3T3 fibroblasts on elastic and viscoelastic gels presenting collagen I on the network of PAA (NHS) or on both types of PAA (SS).

The speed of 3T3 fibroblasts on elastic and viscoelastic gels was monitored for 4 h after the fibroblasts reached the steady state. On PAA gels presenting collagen I on the network, fibroblasts move faster on viscoelastic gels than on elastic gels. The increased cell motility observed on viscoelastic gels presenting collagen I on the network could be attributed to their inability to form focal adhesions, which favored cell displacement.^28^ Cell motility is a subtle interplay between forming adhesions that are strong enough to generate forces that will push the cell forward and weak enough to be disassembled in order to move the cell.^29^ This lack of large focal adhesions prevents the formation of acto-myosin stress fibers^25,27^ but presumably not the formation of branched actin that pushes the membrane forward and initiates cell movement,^30^ thus facilitating locomotion, as there is no need to disassemble large adhesions at the rear end of the cell to move. On gels presenting collagen I on both forms of PAA, the speed of fibroblasts was similar on elastic and viscoelastic gels. We previously demonstrated that in these conditions, 3T3 cells form a similar number of focal adhesions with their substrates on both elastic and viscoelastic gels, but these adhesions are smaller on the viscoelastic substrate.^16^ This observation suggests that adhesions' size has less influence on cell motility than the number of adhesions on soft substrates.

### Viscous dissipation affects the morphology and spreading of epithelial and mesodermal cell types

**FIG. 2.**
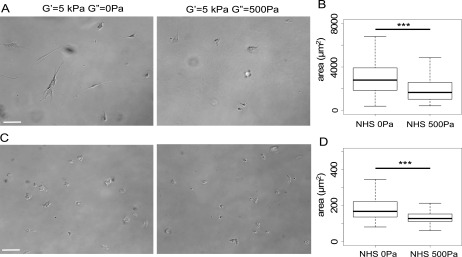
Morphology and projected area of HASM and 22Rv1 cells on 5 kPa viscoelastic and elastic gels. A: Bright field images of HASM cells. Scale bar = 100 *μ*m. B: Projected area of HASM cells after 24 h on 5 kPa elastic and viscoelastic gels coated with collagen I. C: Bright field images of 22Rv1 malignant prostate epithelial cells. Scale bar = 100 *μ*m. B: Projected area of 22Rv1 cells after 24 h on 5 kPa elastic and viscoelastic gels coated with collagen I.

In addition to 3T3 fibroblasts, the responses of human airway smooth muscle (HASM) airway cells and 22Rv1 malignant prostate cells to viscous dissipation have been characterized. Both types of cells were plated on collagen I-coated gels for 24 h, bright field images of these cells were taken [Figs. 2(a) and 2(c)], and their average areas were quantified.

HASM cells are well spread and elongated on 5 kPa elastic gels. Some of the smooth muscle cells are extremely elongated and send projections along the longer cellular axis. On 5 kPa viscoelastic gels, HASM cells are rounder and do not present long cellular projections or an obvious cellular axis. Additionally, their area is significantly smaller on viscoelastic gels than on elastic gels of similar elastic modulus [Fig. 2(b)]. Smooth muscle airway cells have been described to be mechanosensitive,^31^ and their projected area became greater as the storage modulus of the matrix increased.

22Rv1 cells have similar morphologies on 5 kPa elastic and viscoelastic gels [Fig. 2(c)], but they spread significantly less on viscoelastic gels [Fig. 2(d)]. This observation was surprising because 22Rv1 cells have previously been described to be insensitive to the elastic modulus of their substrate.^32^ We speculate that the elastic and the viscous moduli of the substrate might be integrated through different signaling pathways within these cells.

## CONCLUSION

In this article, we describe a method to produce PAA viscoelastic gels for *in vitro* cell culture. The storage and the loss moduli of these gels can be tuned to closely replicate the mechanical properties of biological tissues, at least at small strains. Since these gels are linearly elastic, they do not capture the strain dependence of stiffness reported for some tissues. We demonstrate that cell types from mesenchymal, mesodermal, and epithelial origins are sensitive to the viscous dissipation of their substrate. Thus, viscoelastic PAA gels are a powerful tool to study the role of the mechanical environment, and notably of viscous dissipation, in the phenotype of a wide range of primary or established cell lines. Additionally, soft viscoelastic hydrogels can be tuned to closely mimic the mechanics of different cellular niches and present promising potential to maintain stem cells and primary cells undifferentiated in culture.

## METHODS

PAA viscoelastic gels are made by cross-linking *acrylamid*e and *bis-acrylamide* in the presence of long linear polymers of polyacrylamide.^16^ The quantities of *acrylamid*e and *bis-acrylamide* polymerized into a network control the elasticity of the gel (see examples in Table I). The amount of linear PAA incorporated into the network determines its viscous properties. We formulate different recipes for elastic and viscoelastic gels with storage moduli of 1 and 5 kPa and loss moduli up to 10% of the storage moduli (Table I). Our method also allows selective covalent coupling of adhesion proteins to either the networked or the linear PAA. The rheological characterization of polyacrylamide with and without linear PAA has previously been described in other works,^16,19^ and therefore, Table I presents averaged values of the G′ (elastic) and G″ (viscous) moduli measured at a constant frequency of 1 rad/s. Since the gels are linearly elastic, a wide range of strains can be used to measure G′ and G″. Depending on the sensitivity of the instrument used and the magnitude of G′, a shear strain of 1% to 5% is generally recommended.

**TABLE I.** Averaged values of the elastic and viscous moduli measured at 1 rad/s as a function of the content of *acrylamide*, *bis-acrylamide*, and linear PAA in the initial gel mix. n = 5 gels.

G′ (Pa)	G″ (Pa)	% acrylamide	% bis-acrylamide	% of linear acrylamide
1636	1	4.5	0.1	0
1590	206	5.5	0.1	2.75
5580	10	8	0.1	0
6280	490	8	0.15	2.75

### 1-Prepare coverslips

The gel will be cast between two glass coverslips in order to be flat. Glass coverslips need to be functionalized in order for the gel to stick to the bottom coverslip and to be non-adherent to the top coverslip.

### Glutaraldehyde treatment for the adhesive coverslip

• Immerse coverslips in 0.1 M NaOH for 1 min.
• Remove NaOH and air-dry coverslips.
• Under a chemical hood, immerse coverslips in 3-APTMS for 3 min.
• Wash several times with H_2_0 until no foaming is observed. If 3-APTMS is not removed, it will react with glutaraldehyde in the next step and form an orange precipitate. If an orange precipitate is observed, discard coverslips and restart at step 1.
• Cover each glass with 0.5% glutaraldehyde and let sit 1 h.
• Aspirate off glutaraldehyde and air-dry coverslips (do not rinse).
• Store coverslips in a vacuum container.

### SurfaSil treatment for non-adhesive coverslips

• Dilute SurfaSil Siliconized Fluid (Interchem) in acetone to make a 5% working solution.
• Immerse glass coverslips in SurfaSil solution for 10 s.
• Rinse coverslips with acetone.
• Rinse coverslip with methanol two times. This rinse prevents interaction of the SurfaSil coating with water, thus reversing the siliconization.
• Air-dry coverslips.

### 2-Polymerize the linear PAA

• Make a 5% acrylamide solution in H_2_O according to the recipe in Table II.
• Add TEMED 0.05% and ammonium persulfate (APS) 0.025%. The amount of APS is very low in order to ensure the formation of long linear PAA chains, and so their radii of gyration are much larger than the mesh size of the crosslinked network and the linear chains will not be able to quickly diffuse out of the free surface gel.
• Polymerize for 1h at 37 °C.
• Store at 4 °C in the dark; this solution can be stored for months.
• Linear polyacrylamide solution when properly polymerized is highly viscous, therefore cutting the pipette tip before solution transferring might be needed.

**TABLE II.** Recipes for 10 ml of inert or activated linear PAA solution.

Type of linear PAA	Acrylamide 40%	H_2_O	TEMED	APS	NHS 4% in DMSO
inert	1.25 ml	8.72 ml	5 *μ*l	24 *μ*l	…
activated	1.25 ml	7.72 ml	5 *μ*l	24 *μ*l	1 ml

#### 3- Covalent attachment of adhesion proteins or other ligands to the viscoelastic substrate

The adhesive ligand to which the cell attaches can be linked to the dissipative linear chains alone, to the elastic crosslinked network alone, or both linear and crosslinked PAA of the viscoelastic gel. Separate methods are needed for each of these three ways to attach adhesive ligands.

### Attach proteins to the linear PAA: NHS in the DMSO method

**FIG. 3.**
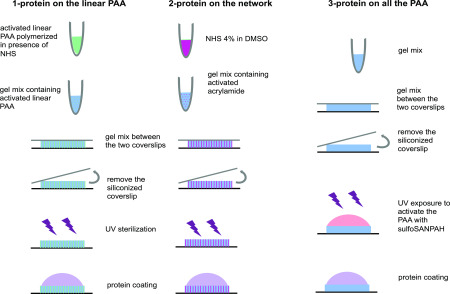
Illustration of the three methods to make viscoelastic gels presenting adhesion proteins on the linear PAA, the network of PAA or both types of PAA.

In order to activate the linear PAA and cross-link proteins to the dissipative part of the gel, which proceeds as described above, add 1 ml of N-hydroxysulfosuccinimide (NHS) 4% in dimethyl sulfoxide (DMSO) to the 5% acrylamide solution and then add TEMED and APS to initiate the polymerization (Fig. 3, left panel). The resulting linear PAA solution will covalently bind proteins upon incubation within a protein solution in HEPES (50 mM at pH = 8.2).

### 3-Polymerize the PAA network

The next step in the method is to polymerize the network to effectively form a hydrogel. The recipes presented in Table I can be modified in order to change the properties of the network; however, when trying a new recipe, make sure that gels do not swell after incubation in cell media. Changes in the gel volume upon immersion can induce changes in their mechanical properties too.

### Make the hydrogel

• Mix acrylamide, bis-acrylamide, linear acrylamide, TEMED, and H_2_O according to the recipe presented in Table III.
• Add APS 10%, mix and pipet 35 *μ*l of gel mix, deposit it on a glutaraldehyde-treated coverslip, and then add a siliconized coverslip on top of the droplet.
• After 15 min, add milliQ H_2_O on the side of the gel to avoid drying.
• After 15 additional minutes, remove the top coverslip and immerse the gel in H_2_O. If there is access to a shear rheometer, the viscoelastic properties of the gel can be directly measured from an analogous sample polymerized between the plates of the rheometer.

**TABLE III.** Gel recipes in *μ*l for the total of 500 *μ*l gel mix. The underlined numbers are for gels made with the NHS in the DMSO method where 50 *μ*l of NHS in 4% DMSO is added to the gel mix.

Gel G′(k Pa), G″(Pa)	Acrylamide 40% stock	Bis-acrylamide 2% stock	H_2_O	TEMED	APS	Linear PAA
1 kPa, 1 Pa	56	25	414	1.25	3.75	…
	56	25	364	1.25	3.75	…
1 kPa, 200 Pa	69	25	80	1.25	3.75	321
	69	25	30	1.25	3.75	321
5 kPa, 10 Pa	100	25	370	1.25	3.75	…
	100	25	320	1.25	3.75	…
5 kPa, 500 Pa	100	37.5	336	1.25	3.75	286
	100	37.5	286	1.25	3.75	286

#### Potential problems and solutions

Because the exact values of G′ and G″ will depend on the quality of the reagents used for gel formulation, it is recommended when it is possible to measure the viscoelasticity directly. In our experience, mixing of linear chains into the polymerizing crosslinked acrylamide is not always identical, and G′ tends to decrease as reagents get old, presumably due to oxidation. The changes can be as large as a factor of 50%.

The main limitation of this viscoelastic gel formulation is the possibility that the linear PAA chains will escape the gel surface, which can occur on long time scales. Therefore, we suggest that the gels are freshly used for cell culture and not stored for weeks prior to experiments. Moreover, our viscoelastic systems can potentially exhibit microphase separation, and the linear chains are free to diffuse out of the free surface of the gel, but at the level of light microscopy using 100× objectives, we see no evidence for significant surface differences, except that macroscopically viscoelastic gels can look slightly cloudy.

### Attach protein to both the linear (1) and the network PAA (2): Sulfo-SANPAH method [Fig. 3 right panel (3)]

• Rinse the gel with 50 mM HEPES, pH = 8.2.
• Prepare a 5 mM sulfosuccinimidyl 6–(4′-azido-2′-nitrophenylamino)hexanoate (sulfo-SANPAH) solution in 75% H_2_O and 25% DMSO.
• Cover the surface of the gel with the sulfo-SANPAH solution and illuminate with a 320–365 nm UV light source for 15 min. Properly activated sulfo-SANPAH should change color from bright orange into darker, burnt orange. Rinse 3 times with HEPES: 50 mM pH = 8.2.
• Immerse the gels in the protein solution in HEPES: 50 mM pH = 8.2. Incubate for 2 h at RT or overnight at 4 °C (both incubations result in uniform surface coating). UV-irradiated sulfo-SANPAH-coated gels should be immersed in the protein solution not later than 30 min postactivation to ensure optimal reactivity.
• Rinse 3 times with PBS and store at 4 °C for up to 2 days before seeding cells.

### Attach proteins to the network: NHS in the DMSO method [Fig. 3 middle panel (2)]

• Prepare NHS solution: 4% in DMSO.
• Add 100 *μ*l of NHS 4% in DMSO for 1 ml of gel mix. Adjust the amount of H_2_O to compensate for the NHS in DMSO solution added to the gel mix.
• Add APS and TEMED to initiate the polymerization and cast the gel as previously described.
• Once the gel is rinsed and immersed in HEPES 50 mM pH = 8.2, illuminate it for 15 min with a deep UV light to sterilize it.
• Immerse the gels in the protein solution in HEPES 50 mM pH = 8.2. Incubate for 2 h at RT or overnight at 4 °C.
• Rinse 3 times with PBS and store at 4 °C for up to 2 days before seeding cells.

### Attach proteins to the network: NHS in the toluene method

It is possible to use a solution of 2% NHS in toluene instead of 4% NHS in DMSO. However, this method is less effective for our gels, notably because the viscosity of the gel mix makes it harder to remove the toluene. Small droplets of toluene often remain in the gel mix and interfere with the polymerization of the network. In this case, follow these steps:

• Add 200 *μ*l of NHS 2% in toluene to 1 ml of gel mix and thoroughly agitate until the solution becomes uniformly turbid. This indicates that there are small droplets of toluene formed within the gel mix solution.
• Incubate for 5 min on the bench and centrifuge at 10 000 × g for 5 min to separate the toluene from the aqueous gel mix.
• Remove the toluene by pipetting the gel mix in the lower layer in the tube and transfer it to a new tube.
• Initiate the network polymerization and follow the same procedure as described in the *NHS in the DMSO* section.

#### Potential problems and solutions

The crucial element in making viscoelastic substrates is the solution of long unbranched polyacrylamide chains needed to add dissipation to the elastic crosslinked PAA network. The chains should be as long as possible and at least several times larger in radius of gyration than the mesh size of the network. For a 5% crosslinked polyacrylamide gel, the mesh size is on the order of 10 nm, and therefore, the radius of gyration of the linear PAA needs to be >40 nm, which requires a very high molecular weight and, therefore, a minimal amount of polymer initiator in order to prevent the rapid formation of numerous short chains. If the chains are too short, they can diffuse out of the network, disturbing the substrate surface and altering the viscoelasticity. At the same time, growing free radical chains need to be protected from quenching by oxygen or other electron-rich molecules in the solution. Care must be taken to carefully de-oxygenate all solutions to prevent termination of the polymerization reaction before the reaction is complete. A third issue is related to the high viscosity of the linear PAA stock solution. Mixing the acrylamide and bis-acrylamide monomer solutions as well as the APS and TEMED needed to initiate network polymerization into the linear PAA must be done not only slowly to prevent air bubbles from forming but also rapidly enough so that the gel point of the network is not reached before mixing is complete. The small volumes (<1 ml) to make substrates suitable for microscopy can be done conveniently using the protocols listed here. Larger volumes might need adjustment to the mixing method or substitution of UV-activated or thermally activated initiators.

### 4- Cell seeding

• 30 min before plating cells, immerse the gel in cell media and incubate at 37 °C. This step ensures that gels are at 37 °C and soaked in media when cells are plated.
• Remove enough media so that gels will be hydrated but not immersed.
• Plate the cells on the gel in a small volume to maximize cellular adhesion.
• After 2 h or once cells start to spread, add media on the plate to immerse the gels and the cells.

### 5- Cell culture

3T3 mouse fibroblasts from ATCC were maintained in DMEM (Gibco) with 10% fetal bovine serum (ATCC) and 1% streptomycin and penicillin (Gibco). 22Rv1 cells from ATCC were cultivated in RPMI (ATCC) with 10% fetal bovine serum (ATCC) and 1% streptomycin and penicillin (Gibco). Human airway smooth muscle (HASM) cells from ATCC were maintained in F-12 media supplemented with 10% fetal bovine serum (ATCC) and 1% streptomycin and penicillin (Gibco). Cells were cultivated at 37 °C and 5% CO_2_ in a humid atmosphere. Cells were passaged with trypsin (Gibco) once they reached confluence. No animal or human studies were performed, and therefore, ethics approval is not required.

### 6- Traction force microscopy

Traction force microscopy can be performed with these gels. In order to monitor the gel deformation, 0.2 *μ*m fluorescent beads are included inside the gel. The gel mix containing beads needs to be thoroughly vortexed to ensure a good dispersion of the beads inside the gels. The gels are then made as described above. To quantify the deformation made by cells, we took one phase image of a cell and two images of fluorescent beads before and after removing the cell from the gel. To ensure an accurate estimation of the gel deformation, we waited for at least 200 s before taking the bead image after removing cells so that the gel underwent a complete relaxation. We first quantified gel deformation using particle image velocimetry^20,21^ and calculated traction using Fourier Transform Traction Microscopy.^21,22^ Cellular contraction was quantified using net contractile moment, which is a scalar measure of cell's contractile strength.^22^ Because the relaxation time of the viscoelastic gel is much shorter than the time for cells to spread and build contractile force,^23^ we used the same home-made codes for both elastic and viscoelastic gels.

### 7- Statistics

The distribution of the projected cell area, spreading velocity, net contractile moment, and motility speed values was tested for normality using the Shapiro–Wilk test. The distribution of our datasets did not follow a Gaussian law, and so we used non-parametric statistics tests to compare cells on elastic and viscoelastic substrates. Kruskal–Wallis and ANOVA with the Tukey post-tests were applied to compare datasets. Differences were considered to be statistically significant when p-values <0.01. The * symbol indicated p-values < 0.01, ** was for p-values < 0.001, and *** for p-values < 0001. Statistically similar datasets were indicated by “ns.”

## Data Availability

The data that support the findings of this study are available from the corresponding author upon reasonable request.

## CODE AVAILABILITY

The home-made Matlab analysis codes can be made available upon request to Jeffrey Fredberg (jjf@harvard.edu)
